# The Evolutionarily Conserved Tre2/Bub2/Cdc16 (TBC), Lysin Motif (LysM), Domain Catalytic (TLDc) Domain Is Neuroprotective against Oxidative Stress*

**DOI:** 10.1074/jbc.M115.685222

**Published:** 2015-12-14

**Authors:** Mattéa J. Finelli, Luis Sanchez-Pulido, Kevin X Liu, Kay E. Davies, Peter L. Oliver

**Affiliations:** From the MRC Functional Genomics Unit, Department of Physiology, Anatomy, and Genetics, University of Oxford, Parks Road, Oxford OX1 3PT, United Kingdom

**Keywords:** evolution, mouse, neurobiology, neurodegeneration, neurological disease, neuron, oxidative stress, protein structure

## Abstract

Oxidative stress is a pathological feature of many neurological disorders; therefore, utilizing proteins that are protective against such cellular insults is a potentially valuable therapeutic approach. Oxidation resistance 1 (OXR1) has been shown previously to be critical for oxidative stress resistance in neuronal cells; deletion of this gene causes neurodegeneration in mice, yet conversely, overexpression of OXR1 is protective in cellular and mouse models of amyotrophic lateral sclerosis. However, the molecular mechanisms involved are unclear. OXR1 contains the Tre2/Bub2/Cdc16 (TBC), lysin motif (LysM), domain catalytic (TLDc) domain, a motif present in a family of proteins including TBC1 domain family member 24 (TBC1D24), a protein mutated in a range of disorders characterized by seizures, hearing loss, and neurodegeneration. The TLDc domain is highly conserved across species, although the structure-function relationship is unknown. To understand the role of this domain in the stress response, we carried out systematic analysis of all mammalian TLDc domain-containing proteins, investigating their expression and neuroprotective properties in parallel. In addition, we performed a detailed structural and functional study of this domain in which we identified key residues required for its activity. Finally, we present a new mouse insertional mutant of *Oxr1*, confirming that specific disruption of the TLDc domain *in vivo* is sufficient to cause neurodegeneration. Our data demonstrate that the integrity of the TLDc domain is essential for conferring neuroprotection, an important step in understanding the functional significance of all TLDc domain-containing proteins in the cellular stress response and disease.

## Introduction

Oxidative stress arises from the accumulation of reactive oxygen species (ROS)^2^ generated by oxygen metabolism that can in turn lead to lipid, protein and DNA damage, and ultimately cell death (1–3). Significantly, oxidative stress is implicated in many progressive neurodegenerative disorders, including Alzheimer disease, Parkinson disease, and amyotrophic lateral sclerosis (ALS), yet the exact role of this process in neuronal cell death is still unclear (4, 5). Given the importance of oxidative stress in many disease states, it has been suggested that proteins that counteract or reduce the levels of ROS in cells could be employed for therapeutic benefit (2, 6).

The oxidation resistance 1 (*OXR1*) gene was originally identified in a screen conducted in *Escherichia coli* for genes that were able to protect cells against oxidative damage (7). We and others demonstrated subsequently that modulating the levels of OXR1 can influence the cellular response to oxidative stress (8–12) and that loss or disruption of OXR1 or its orthologues *in vivo* results in early lethality and selective neurodegeneration (8, 11, 13). Furthermore, we went on to show that overexpression of Oxr1 could delay pathogenesis in a mouse model of ALS (14) as well as restore the cellular defects associated with familial ALS mutations (15). Despite these findings, the function of OXR1 remains unknown. It is noteworthy, however, that OXR1 contains a conserved protein motif of ∼200 amino acids named the TLDc domain (16–18). Moreover, OXR1 is expressed as several isoforms that all contain this C-terminal region, and it has been shown that even the shortest of these isoforms functionally compensates for the full-length protein *in vitro* (8). This suggests that it is the TLDc domain that plays a key role in oxidative stress resistance.

In mammals the TLDc domain is also found in a family of proteins including nuclear receptor coactivator 7 (NCOA7), TBC1D24, KIAA1609 (also referred to as TLDC1), and C20ORF118 (or TLDC2) (19). In addition to studies on OXR1, TBC1D24 is associated with a range of severe human neurological disorders. An increasing series of compound heterozygous and homozygous TBC1D24 coding and frameshift mutations has been identified, including mutations in the TLDc domain itself, which cause diverse forms of epileptic syndromes including familial myoclonic epilepsy (20, 21, 25), familial malignant migrating partial seizures of infancy (22), early infantile epileptic encephalopathy (27), deafness, onychodystrophy, osteodystrophy, and mental retardation syndrome (DOORS) (23), neurodegeneration (25), non-syndromic hearing impairment (26, 27), focal epilepsy associated with intellectual disabilities (24, 25), and more recently, multifocal myoclonus involving cerebellar dysfunction (28). Initial functional studies of TBC1D24 have focused on the TBC domain, suggesting that mutant forms of the protein influence cortical maturation and migration via control of GTP-binding protein ADP-ribosylation factor 6 (ARF6) activation (21, 29) as well as vesicle recycling in neurons (30, 31). Taken together, these data suggest that OXR1 and TBC1D24 are important for normal brain function. NCOA7 is also expressed in the brain (32), although nothing is known regarding its transcriptional role in the nervous system. To assist these studies, the three-dimensional structure of the TLDc domain has been solved (17). Nevertheless, due in part to the lack of structural similarity to known protein folds, it remains unclear whether other TLDc proteins possess similar functional properties and how the TLDc domain contributes to essential cellular processes; indeed, there is no evidence that the TLDc domain imparts catalytic properties as originally predicted (18).

To, therefore, understand the functional significance of this domain, we carried out a structural-functional study of all mammalian TLDc proteins, demonstrating that they share a common protective role in neurons and identifying critical residues in the domain. Our data provide valuable new insight into the function of TLDc domain-containing proteins in the neuronal stress response.

## Experimental Procedures

### 

#### 

##### In Situ Hybridization

Regions representing all isoforms of *Oxr1* (2723–3139 bp of NCBI accession number NM_130885), *Ncoa7* (2650–3284 bp of NM_172495), *Tbc1d24* (2705–3558 bp of NM_001163847), *Kiaa1609* (1337–1959 bp of NM_028883), and *C20Orf118* (231–620 bp of NM_001177439) were amplified by RT-PCR or PCR and cloned into pCR4-TOPO (Invitrogen). Riboprobe synthesis and slide hybridization was carried out as previously described (33). All slides shown were developed for 16 h.

##### Quantitative Real-time PCR (qRT-PCR)

Total RNA was extracted from Neuro 2a (N2a) cells using an RNeasy mini kit (Qiagen) and reversed-transcribed (ThermoScientific). Subsequent qRT-PCR was performed in an ABI PRISM 7000 sequence detection system (Applied Biosystems) using SYBR Green PCR master mix (Applied Biosystems) and primers shown in Table 1. The housekeeping gene *Gapdh* was used as an internal normalizing control, and the ΔΔCt method was used to calculate the level of mRNA -fold changes, or the mean Ct value of each TLDc gene was compared with the mean Ct value for *C20Orf118*.

**TABLE 1:** **List of primers used for qRT-PCR** F, forward; R, reverse.

Primer	Sequence 5′–3′
Gapdh F	AGAACATCATCCCTGCATCC
Gapdh R	CACATTGGGGGTAGGAACA
Oxr1FL F	CAGTCGTGACTGGACAGGTTT
Oxr1FL R	ATGGGCTACATCTGGAGTCG
Oxr1 C F	CCATAAATACACTCTGGTAGTGTCG
Oxr1 C R	TTTGGTCGGAAAGATTCAGG
Ncoa7 FL F	TGTCGCTACTTCACTGATGG
Ncoa7 FL R	GCGTCTTTGATCTTCATGTG
Ncoa7 B F	GCCCCTGGACATTCAGATT
Ncoa7 B R	CTGTGGGGCTGTAGGATAGG
Tbc1d24 F	AATGGCCAATGAGAAAGCA
Tbc1d24 R	AGGGATCCAGGACCAAATG
Kiaa1609 F	GCAGGCTGAGGTAGACAAG
Kiaa1609 R	CCCTCTGCATGCCGTTATAC
C20Orf118 F	TCAGCTTACAGAAGCTAGCC
C20Orf118 R	CTGTCCATCCTGATCTCTGA

##### In Silico Analysis

Alignments were produced with T-Coffee (34) using default parameters and refined manually. Protein alignments are presented using Belvu (35) with a coloring scheme indicating the average BLOSUM62 scores (which are correlated with amino acid conservation) of each alignment column: red (>3.5), purple (between 3.5 and 2), and light yellow (between 2 and 0.5). Sequences were named with their Uniprot identifiers. The corresponding secondary structures were acquired from the zebrafish OXR2-TLDc domain structure (PDB ID 4ACJ) (17). The NCOA7-B structural model was created using Modeler (36) based on the zebrafish OXR2-TLDc domain structure (PDB ID 4ACJ) (17). The NCOA7-B-TLDc domain model is presented using PyMOL. The amino acid evolutionary conservation was based on alignment BLOSUM62 scores.

##### Constructs and Mutagenesis

Full-length cDNAs representing mouse *Ncoa7*, *Tbc1d24*, *Kiaa1609*, and *C20Orf118* (accession numbers as above) and short isoform *Ncoa7-B* were cloned into a pcDNA3 vector with an HA tag in-frame at the C-terminus. Oxr1-FL and Oxr1-C have been described previously (8). For *Ncoa7*, *Tbc1d24*, and *Kiaa1609* knockdown experiments, shRNA constructs were purchased from Sigma (NM_172495.5–2897s21c1, NM_173186.2–1208s1c1, and NM_028883.2–256s1c1, respectively). Site-directed mutagenesis was used to introduce the various mutations into the pcDNA3 constructs above as per the manufacturer's protocol (Agilent Technologies) and confirmed by sequencing.

##### Cell Culture, Transfection, and Treatment

Neuronal N2a cells were cultured in DMEM supplemented with glutamax (Gibco), 1% penicillin-streptomycin (Gibco), and 10% fetal calf serum (Gibco). Cells were transfected with either FuGENE 6 (Promega) for 48 h (for shRNA experiments and overexpression for lipid peroxidation and *S*-nitrosylation assays) or Magnetofection (OZBiosciences) for 24 h (for all other overexpression experiments) as per the manufacturers' protocol. Cells were treated with arsenite (50 μm for 5 h to quantify ROS, 50 μm for 4 h lipid peroxidation and *S*-nitrosylated assays, or 150 μm for 4 h to quantify cell death) in water or with water-only as a control. To visualize pyknotic nuclei, cells were fixed with 4% paraformaldehyde for 10 min at room temperature, washed with PBS twice, blocked with blocking buffer (5% goat serum, 0.5% Triton X-100) for 1 h at room temperature, and mounted in DAPI medium (Vectorlabs). Cells were visualized using a fluorescent microscope (Leica), and those with typical condensed and fragmented nuclei were counted as pyknotic cells. Anti-HA immunostaining was carried out as previously described (15).

##### Purified Cortical Cell Cultures

Cortical cells from wild-type P1 pups were prepared and cultured as previously described (37). Briefly, cortices were dissected in cold Hanks' balanced salt solution (Gibco), and meninges and hippocampi were removed and trypsinized for 15 min at 37 °C. Trypsin was inactivated using trypsin inhibitor (Life Technologies) for 5 min, and cortices were triturated and purified on an OptiPrep gradient following Brewer and Torricelli (37). Cells were plated in culture medium (Neurobasal phenol-free, 5% FCS, 2% B27, 0.5 mm l-glutamine, Gibco) and cultured for 5 days before treating with vehicle or arsenite (0.5 μm for 2 h).

##### Dihydroethidium Assay

Cells were cultured in 96-well plates as described above. Cells were treated for 5 h with arsenite (50 μm) or vehicle supplemented with dihydroethidium (Sigma) diluted in culture medium (15 μm). After experimental treatment, cells were washed twice in PBS, wells were filled with 100 μl PBS, and the fluorescence was immediately measured on a Fluostar Omega (BMG Labtech) plate reader at an excitation wavelength of 430 nm and an emission wavelength of 590 nm.

##### Lipid Peroxidation Assay

N2a cells were transfected for 48 h and treated with 50 μm arsenite for 4 h before the levels of lipid peroxidation were quantified using a lipid peroxidation assay as per the manufacturer's protocol (Abcam). Briefly, one confluent 63-cm^2^ dish was used per condition, and cells were washed with cold PBS, homogenized by sonication in the lysis buffer provided, and clarified by centrifugation. Three volumes of thiobarbituric acid were added to the samples and incubated for 1 h at 95 °C. Samples containing a known concentration of malondialdehyde were processed in parallel and used to generate a standard curve. Samples were transferred to a transparent 96-well plate, and absorbance was read in duplicate at 532 nm on a FluostarOmega (BMG Labtech) plate reader.

##### S-Nitrosylated Protein Assay

N2a cells were transfected for 48 h on coverslips and treated with 50 μm arsenite for 4 h, and the levels of *S*-nitrosylated proteins were assessed by using a S-NO protein detection kit (Cayman). Cells were fixed with 4% paraformaldehyde for 20 min at room temperature and subsequently washed and blocked using buffers provided following the manufacturer's protocol. Cells were incubated with reducing and labeling reagents for 1 h at room temperature followed by incubation for an additional hour with fluorescein detection reagent. Cells were observed by fluorescence microscopy (Leica). All images were captured using Axiovision software. The signal intensity of each cell was measured using ImageJ software and expressed as the mean gray value divided by the surface of the cell. Approximately 40 cells were assessed per condition.

##### Tissue Staining

Terminal deoxynucleotidyltransferase dUTP nick end labeling (TUNEL) staining was carried out on fresh frozen brain sections as recommended by the manufacturer (*In situ* cell death kit, Roche Applied Science). For quantification, four 12 μm sections at 60 μm intervals across the midline of the cerebellum were counted for TUNEL-positive cells per replicate. β-Galactosidase staining was also carried out on fresh frozen brain sections as previously described (38).

##### Western Blotting

Protein extracts were prepared from cells using standard radioimmunoprecipitation assay buffer, and protein levels were quantified using BCA assays (Millipore). Proteins were run on pre-cast NuPAGE Bis-Tris gels (Life Technologies) and transferred as per the manufacturer's protocol. Primary antibodies used were as follows: Ncoa7 (Abcam ab103993), Oxr1 (antiserum as previously described (8)), Tbc1d24 p14 (Santa Cruz), Kiaa1609 (Biorbyt), and β-actin (Abcam).

##### Animals

All experiments were conducted in adherence to the guidelines set forth by the UK Home Office regulations and with the approval of the University of Oxford Ethical Review Panel. The *Oxr1* “knock-out first” allele (*Oxr1*^tm1a(EUCOMM)Wtsi^) was generated as part of the International Knock-out Mouse Consortium (IKMC) program (IKMC project 84243). Briefly, a L1L2_Bact_P cassette was inserted after exon 12 of Oxr1, composed of a splice acceptor site and lacZ followed by neomycin under control of the human β-actin promoter (see Fig. 6 and Ref. 39). Thus the lacZ reporter is designed to be under the control of the native promoter of Oxr1-FL and Oxr1-C. Intercrossing of mice heterozygous for the tm1a allele was used to generate homozygous animals, thus maintaining the line on a C57BL/6N background.

##### Statistical Analysis

Prism GraphPad software was used for one-way ANOVA with Dunnett's multiple comparison test or *t* tests. Quantified data are presented ±S.E.

## Results

### 

#### 

##### TLDc Domain-containing Genes Show Distinct Tissue Distribution Patterns

**FIGURE 1.**
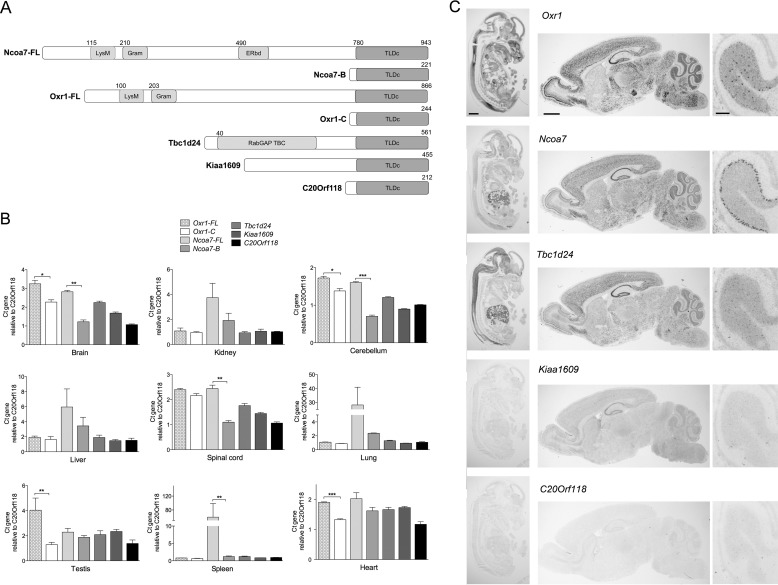
**The TLDc members have specific expression patterns in various organs in embryonic and adult wild-type mice.** *A*, a schematic of the TLDc family members indicating their respective unique and shared domains. *LysM*, lysin motif; *GRAM*, GRAM domain; *ERbd*, estrogen receptor binding domain. Numbers indicate amino acid positions in the corresponding mouse protein. *B*, qRT-PCR of TLDc domain-encoding genes from the tissues indicated from male mice. Data are shown relative to expression of *C20Orf118* and are represented as the mean ± S.E. *, *p* < 0.05; **, *p* < 0.01; ***, *p* < 0.001; *t* test. *C*, *in situ* hybridization of TLDc domain-containing genes in the embryo (E14.5, *left panel*), adult brain (*middle panel*), or adult cerebellum (*right panel*) from wild-type male mice. Riboprobes were designed to span all isoforms of each gene. *Scale bars*: 1 mm (*left* and *middle panel*) and 200 μm (*right panel*).

To compare systematically the expression pattern of all the TLDc domain-encoding genes in parallel, we quantified their mRNA levels in a range of organs from wild-type mice. For *Oxr1* and *Ncoa7* we assayed the full-length transcripts (*Oxr1-FL* and *Ncoa7-FL*) in addition to the shortest known isoforms that almost exclusively contain the TLDc domain, *Oxr1-C* (9, 15) and *Ncoa7-B* (40) (equivalent to human *NCOA7-AS*) (41) (Fig. 1*A*). We showed that *Oxr1-FL*, *Oxr1-C*, *Ncoa7-FL*, and *Tbc1d24* are the most highly expressed TLDc genes in the central nervous system (Fig. 1*B*). In addition, both *Ncoa7* isoforms tested are more highly expressed in the kidney, liver, lung, and spleen than other TLDc genes, suggesting that *Ncoa7* has a specific function in these peripheral organs (Fig. 1*B*). Interestingly, the relative expression of *Ncoa7-FL* and *Oxr1-FL* is significantly higher than their corresponding short isoforms in several tissues, supporting the notion that these specific isoforms are differentially or independently regulated (Fig. 1*B*). We next used *in situ* hybridization to determine the anatomical localization of the TLDc domain-encoding genes during development at embryonic day (E) 14.5 using riboprobes spanning exons common to all known isoforms. These data show that *Oxr1* and *Ncoa7* are expressed throughout the developing embryo, whereas *Tbc1d24* expression is more localized to the brain, spinal cord, and liver (Fig. 1*C*). *Kiaa1609* and *C20orf118* were, however, virtually undetectable at this developmental time point (Fig. 1*C*). Lastly, we investigated the localization of the TLDc-encoding genes in the adult brain, and we showed that *Ncoa7*, *Oxr1*, and *Tbc1d24* are expressed in all major structural regions, particularly in the cortex, hippocampus, and cerebellum, with *Ncoa7* expression enriched in Purkinje cells as opposed to the cerebellar granule cell layer for *Oxr1* and *Tbc1d24* (Fig. 1*C*). In agreement with our qRT-PCR data, *Kiaa1609* is expressed at a generally low level, although expression in the hippocampus is apparent (Fig. 1*C*).

##### All TLDc Proteins Are Able to Confer Protection against Oxidative Stress

**FIGURE 2.**
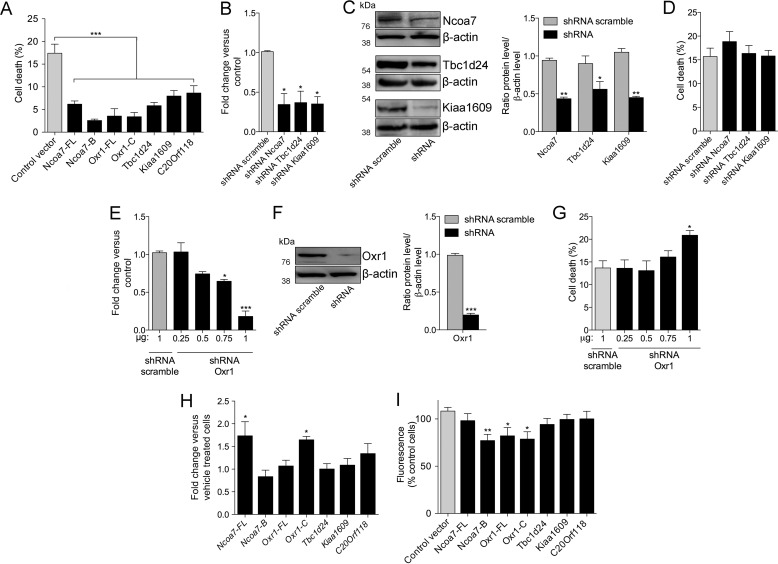
**The TLDc members are neuroprotective against oxidative stress.** *A*, *D*, and *G*, N2a cells were transfected with the constructs indicated and treated with 150 μm arsenite for 4 h. Cell death was quantified by counting the number of cells with pyknotic nuclei. *A*, overexpression of all TLDc proteins significantly reduces the proportion of cell death compared with transfection of a control empty vector. *B*, levels of *Ncoa7*, *Tbc1d24*, and *Kiaa1609* were significantly knocked down in shRNA-transfected N2a cells compared with shRNA scramble-transfected cells. *C*, representative Western blots and quantification showed that the same shRNA constructs significantly reduced protein expression levels. *D*, no significant difference in cell death occurred in *Ncoa7*, *Tbc1d24*, and *Kiaa1609* knockdown cells compared with the control shRNA. Increasing concentrations of an shRNA against *Oxr1* leads to increasing knockdown at the RNA and protein level (*E*) as shown by a representative Western blots and quantification (*F*). *G*, knockdown of *Oxr1* by 82% led to a significant increase in cell death under oxidative stress. *H*, the mRNA levels of TLDc members under oxidative stress were assessed by qRT-PCR on RNA extracted from purified primary cortical neurons cultured for 5 days and treated with 0.5 μm arsenite for 2 h. The *Ncoa7-FL* and *Oxr1-C* transcripts are significantly induced by arsenite treatment compared with vehicle-treated cells. *I*, N2a cells overexpressing each one of the wild-type TLDc proteins were treated with 50 μm arsenite for 5 h. The level of cellular oxidative stress was quantified using the fluorescent dihydroethidium and compared with the level of fluorescence in cells transfected with empty control vector. Data are represented as the mean ± S.E. *, *p* < 0.05; **, *p* < 0.01; ***, *p* < 0.001, one-way ANOVA with Dunnett's multiple comparison test or *t* test).

Because Oxr1 is known to play a role in the oxidative stress response, we sought to determine whether other TLDc proteins, despite their contrasting sizes and additional domains, share any of these important functional characteristics. To assess this we first quantified the cell viability of neurons transfected with either full-length TLDc constructs or Ncoa7-B or Oxr1-C under arsenite treatment, a well-characterized oxidative stress inducer. Interestingly, we found that expression of every TLDc domain-containing protein significantly reduced the proportion of dying neurons as compared with cells transfected with an empty control vector (Fig. 2*A*). All TLDc proteins reduced the proportion of cell death by at least half, and Ncoa7-B appeared to be the most potent neuroprotective protein in this assay, reducing cell death by up to 85% as compared with control cells (Fig. 2*A*). There was no difference in the levels of cell death between untreated cells expressing the TLDc constructs or the control vector, confirming that overexpressing these proteins was not toxic to the cells (data not shown).

We showed in a previous study that knockdown of *Oxr1* led to increased neuronal sensitivity to oxidative stress *in vitro* (8); thus we investigated whether similar effects would be observed for other TLDc proteins. We were able to knock down *Ncoa7*, *Tbc1d24*, and *Kiaa1609* in N2a cells using shRNAs by 64, 54, and 64% at the RNA level, respectively (Fig. 2*B*), and ∼50% at the protein level (Fig. 2*C*). Treatment of these cells with arsenite led to a small but non-significant increase in cell death compared with those transfected with a control scramble shRNA vector (Fig. 2*D*). To compare these findings with *Oxr1*, we knocked down this gene by as much as 82% of the normal RNA level using increasing concentrations of transfected shRNA (Fig. 2*E*) and showed a corresponding 80% decrease at the protein level (Fig. 2*F*). Interestingly, only at the 82% mRNA knockdown level was there a significant increase in cell death under the same oxidative stress conditions (Fig. 2*G*). These data suggest that reducing the expression levels of TLDc genes to around two-thirds that of their normal level is not sufficient to elicit a change in oxidative stress sensitivity.

It has also been suggested that the levels of OXR1 can be induced as part of the oxidative stress response (9); hence, we next determined whether other TLDc proteins share the same properties by quantifying their mRNA levels in purified primary cortical neurons treated with arsenite. *Ncoa7-FL* and *Oxr1-C* were induced significantly by this treatment, whereas the levels of the other TLDc genes remained unchanged (Fig. 2*H*), suggesting that *Ncoa7-FL* and *Oxr1-C* are more likely to participate in the oxidative stress response under arsenite treatment.

To provide some mechanistic insight into how overexpression of TLDc proteins could be neuroprotective, we tested whether it might occur by reducing the oxidative stress level in cells. Thus we quantified the level of ROS using the fluorescent dye dihydroethidium in N2a cells transfected with each of the wild-type TLDc constructs and then treated with arsenite. We showed that overexpression of Oxr1-FL, Oxr1-C, and Ncoa7-B significantly decreased the level of cellular oxidative stress, whereas the other TLDc proteins caused only a small reduction without reaching significance (Fig. 2*I*). Taken together, our data show that all of the TLDc domain-containing proteins can be neuroprotective, yet Ncoa7-FL, Tbc1d24, Kiaa1609, and C20Orf118 were unable to reduce the level of oxidative stress in treated cells. Therefore, our results indicate that the TLDc domain can elicit some of its functional effects alone, yet the context of this domain among the additional protein motifs in each protein may influence these properties.

##### The TLDc Domain Contains Highly Conserved Residues Required for Its Neuroprotective Function

**FIGURE 3.**
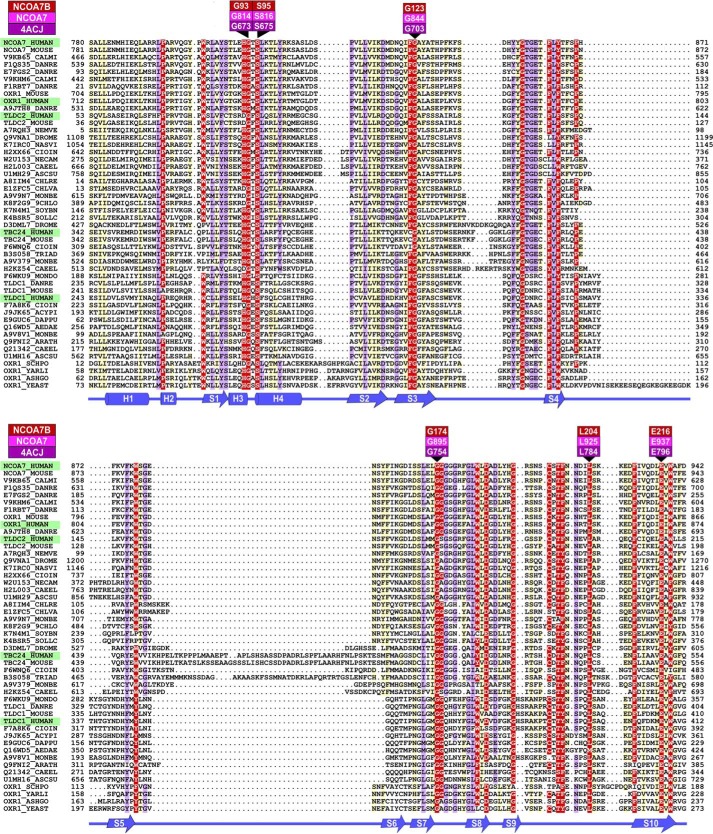
**The TLDc domain is highly conserved across species.** Alignment of 48 TLDc domain-containing proteins. Colors corresponds to the amino acid conservation of each alignment column: *red* (highly conserved), *purple* (mildly conserved), and *light yellow* (poorly conserved based on BLOSUM62 scores). Residues mutated in this study are labeled according to reference protein sequences for human NCOA7-B (*red*), human NCOA7-(FL) (*magenta*), and the zebrafish OXR2-TLDc sequence (PDB ID 4ACJ, *purple*) (17). The limits of the protein sequences included in the alignment are indicated by flanking residue positions. Protein sequences are shown by name or by Uniprot identifier. Secondary structure annotation (in *blue*) is derived from the zebrafish OXR2-TLDc domain (17). α-Helices (*H*) and β-strands (*S*) are indicated by *cylinders* and *arrows*, respectively. Species abbreviations: *9CHLO*, *Bathycoccus prasinos*; *ACYPI*, *Acyrthosiphon pisum* (pea aphid); *AEDAE*, *Aedes aegypti* (yellow fever mosquito); *ARATH*, *Arabidopsis thaliana*; *ASCSU*, *Ascaris suum* (pig roundworm); *ASHGO*, *Ashbya gossypii*; *CAEEL*, *C. elegans*; *CALMI*, *Callorhinchus milii* (ghost shark); *CHLRE*, *Chlamydomonas reinhardtii*; *CHLVA*, *Chlorella variabilis*; *CIOIN*, *Ciona intestinalis*; *DANRE*, *Danio rerio* (zebrafish); *DAPPU*, *Daphnia pulex* (water flea); *DROME*, *Drosophila melanogaster* (fruit fly); *HUMAN*, *Homo sapiens*; MONBE, *Monosiga brevicollis* (choanoflagellate); *MONDO*, *Monodelphis domestica* (opossum); *MOUSE*, *Mus musculus*; *NASVI*, *Nasonia vitripennis* (wasp); *NECAM*, *Necator americanus* (human hookworm); *NEMVE*, *Nematostella vectensis* (starlet sea anemone); *SCHPO*, *S. pombe*; *SOLLC*, *Solanum lycopersicum* (tomato); *SOYBN*, *Glycine max* (soybean); *TRIAD*, *Trichoplax adhaerens*; *YARLI*, *Yarrowia lipolytica*; *YEAST*, *Saccharomyces cerevisiae*.

Next, we investigated how the neuroprotective function of the TLDc proteins may be driven by the conserved TLDc domain. Initially, we performed a protein alignment of this region using sequences from humans to yeast (*Schizosaccharomyces pombe*) to assist in the identification of the most conserved amino acids and structural features (Fig. 3). These analyses predicted 4 α-helices and 10 β-strands and identified ∼10 residue positions within the domain that were highly conserved in all species analyzed (Fig. 3). We hypothesized that these particular residues would be essential for the function of this domain and used an alanine-scanning approach, mutating six of the most conserved amino acids of Ncoa7-B, to investigate the functional consequences. We chose to focus on Ncoa7-B as it is almost entirely composed of the TLDc domain (40), it is expressed from an independent promoter in mammalian cells (41), and it represented the most potent neuroprotective TLDc domain-containing protein we tested in neurons (Fig. 2*A*).

**FIGURE 4.**
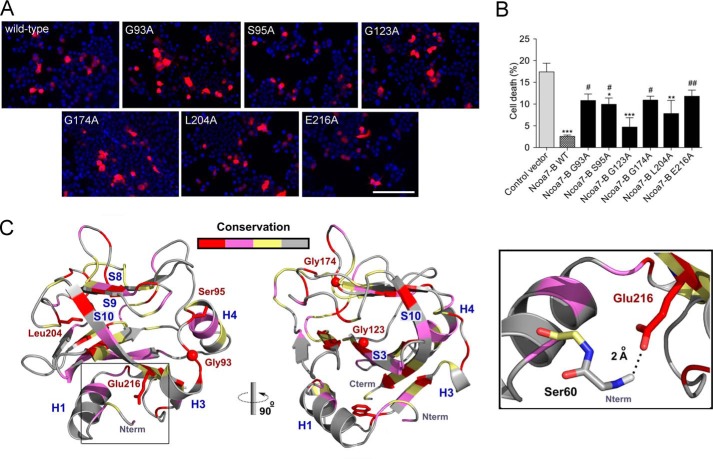
**The TLDc domain reduces levels of oxidative stress in neurons.** *A*, N2a cells were transfected with HA-tagged Ncoa7-B wild-type or mutant followed by HA staining to visualize Ncoa7-B localization. All mutants were expressed, and their localization was similar to the cytoplasmic localization of Ncoa7-B wild type. *Scale bar*: 100 μm. *B*, cell death was quantified by counting the number of pyknotic nuclei of N2a cells transfected with wild-type or mutant Ncoa7-B under oxidative stress and treated with 150 μm arsenite for 4 h. Cell death was compared with levels in cells transfected with control vector (*, *p* < 0.05; **, *p* < 0.01; ***, *p* < 0.001) or with Ncoa7-B WT (#, *p* < 0.05; ##, *p* < 0.01). Data are represented as the mean ± S.E. using one-way ANOVA with Dunnett's multiple comparison test. *C*, the NCOA7-B mutants were mapped onto the zebrafish OXR2-TLDc domain structure (PDB ID 4ACJ) (17) with mutated residues labeled in *red*, side chains as *sticks*, and glycines as *balls*. The ribbon color indicates amino acid evolutionary conservation: *red* (highly conserved), *purple* (mildly conserved), and *light yellow* (low conservation).

We first confirmed that all Ncoa7-B mutants could be expressed efficiently in N2a cells and that the mutations did not affect the cytoplasmic localization of Ncoa7-B (Fig. 4*A*). Next, to assess how these mutations influence the neuroprotective function of the TLDc domain, we quantified cell survival under oxidative stress. Expression of three Ncoa7-B mutants led to a significant reduction in cell death compared with the control vector, suggesting that these mutations were not highly detrimental to Ncoa7-B function (Fig. 4*B*). Interestingly, however, three of the mutants failed to cause the same reduction in cell death and, therefore, appeared to be the most functionally disrupted: G93A, G174A, and E216A (numbering based on Ncoa7-B protein sequence NP_001104737) (Fig. 3).

**FIGURE 5.**
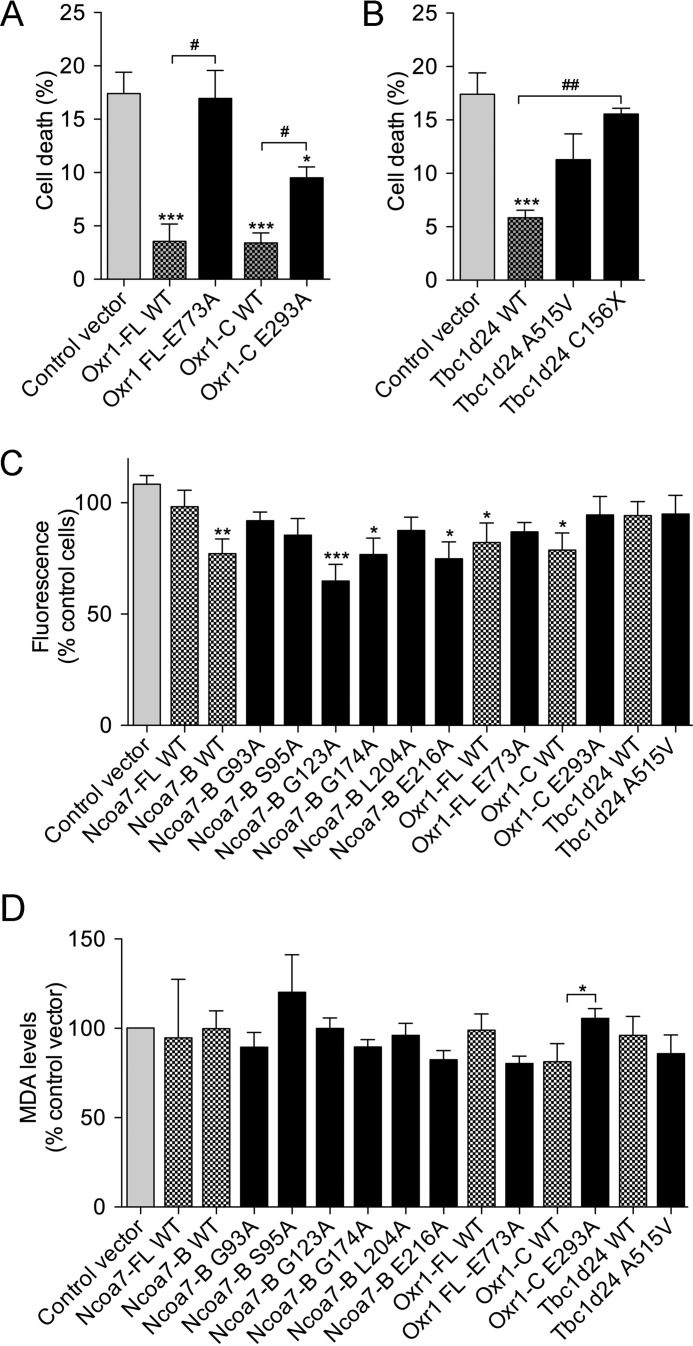
**Specific residues in the TLDc domain are necessary to confer neuroprotection.** *A–D*, N2a cells were transfected with either wild-type or mutant Oxr1, Ncoa7, or Tbc1d24 as indicated and treated with 150 μm arsenite for 4 h (*A* and *B*) or 50 μm arsenite for 5 h (*C*) or 4 h (*D*). *A* and *B*, cell death was assessed by counting the number of pyknotic nuclei compared with cells transfected with the control empty vector (***, *p* < 0.001; *, *p* < 0.05) or the corresponding wild-type construct (##, *p* < 0.01; #, *p* < 0.05). *C*, the level of cellular oxidative stress was quantified using the fluorescent dihydroethidium and compared with the level of fluorescence in cells transfected with empty control vector. *D*, the level of lipid peroxidation (malondialdehyde (*MDA*)) was quantified using fluorescein fluorescence compared with cells transfected with the corresponding empty (*, *p* < 0.05; **, *p* < 0.01; ***, *p* < 0.00) or the wild-type vector (#, *p* < 0.05; ##, *p* < 0.01). Data are represented as the mean ± S.E. using one-way ANOVA with Dunnett's multiple comparison test or *t* test).

We then predicted the consequences of mutating these four specific amino acids on the structure of the TLDc domain of Ncoa7-B using the published zebrafish three-dimensional structure (17) (Fig. 3). We predicted that Gly-93 is likely to be required to maintain the position of α-helices 3 and 4 (42) (Fig. 4*C*). This suggests that the structural integrity of the superficial helix may be important for function of the TLDc domain. Glu-216 is one of the most evolutionarily conserved positions in the TLDc domain, and structural predictions suggest that a hydrogen bond between the carboxylate group of Glu-216 and the amide group of Ser-60 maintains the position of the N-terminal region of the TLDc domain (Fig. 4*C*). Because this particular position seemed likely to influence an important structural feature of the TLDc domain, we extended the alanine scanning to examine the same glutamic acid in Oxr1 using the oxidative stress assay; in both Oxr1-FL (E773A) and Oxr1-C (E293A). The Oxr1-C mutation led to an approximate 2-fold increase in the proportion of cell death compared with wild-type Oxr1-C, suggesting that altering this amino acid was functionally disruptive, as observed for Ncoa7-B. What was more striking, however, was the same mutation in Oxr1-FL almost completely abolished the protective activity of the protein (Fig. 5*A*), further illustrating how the context of the TLDc domain within a larger protein structure may impart different functional properties.

Finally, we assessed mutations in the TLDc domain relevant to epilepsy by testing a Tbc1d24 A515V mutant (equivalent to human A509V) (21) as well as a truncation mutant (Cys156X) to mirror a TBC1D24 frameshift mutation that results in the loss of the TLDc domain (22). Oxidative stress response assays demonstrated that the A515V mutation caused a small but non-significant reduction in cell death compared with a control vector, suggesting that disrupting the disease-associated alanine residue may not have a major influence on the neuroprotective properties of TBC1D24 (Fig. 5*B*). However, the level of neuroprotection provided by the C156*X* mutant was as low as that of the control vector or the Oxr1-FL E773A construct (Fig. 5*B*), suggesting that mutating the highly conserved C-terminal glutamic acid can be as detrimental to the neuroprotective properties of a TLDc protein as losing the entire TLDc domain.

To relate these findings to oxidative stress levels, we went on to measure ROS levels in N2a cells expressing each of the mutant constructs (Fig. 5*C*). These data suggest that there is some correlation between the levels of ROS and how functionally disruptive the mutations were in the cell survival assay (Fig. 5*C*). For example, expressing the helix-associated mutants (G93A) did not significantly reduce cell death or ROS, and the same outcome was observed for the Oxr1-FL E773A mutant (Fig. 5*C*). Conversely, the least destabilizing mutation in Ncoa7-B, G123A, resulted in significantly lower ROS levels, equivalent to cells expressing wild-type Ncoa7-B (Fig. 5*C*). Taken together, these data show that conserved amino acids at key structural positions in the TLDc domain are required to maintain its functional properties in oxidative stress resistance.

Lipid peroxidation and protein *S*-nitrosylation are the two main consequences of increased cellular ROS, and these processes have been implicated in triggering neurodegeneration (2). Thus, we first assessed whether overexpression of TLDc proteins would reduce the level of lipid peroxidation in arsenite-treated N2a cells. No differences in peroxidation were observed between cells transfected with either the empty control vector or any of the wild-type TLDc constructs (Fig. 5*D*). Interestingly, cells overexpressing a mutant version of Oxr1-C (E293A) shown to be functionally disruptive (Fig. 5*A*) caused a significant increase in peroxidation compared with those transfected with the wild-type Oxr1-C vector (Fig. 5*D*). None of the other mutants affected the global levels of lipid peroxidation in transfected cells however.

**FIGURE 6.**
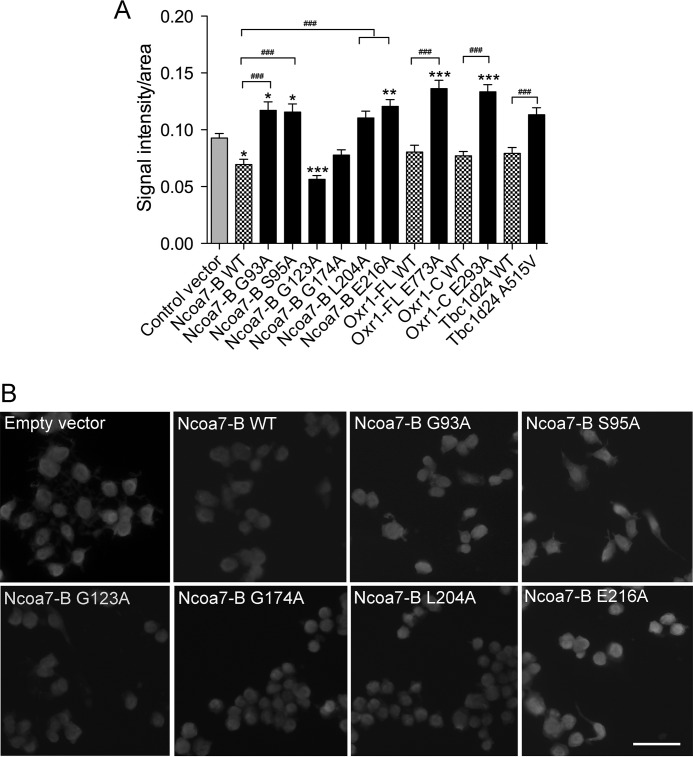
**The TLDc domain is required to reduce levels of *S*-nitrosylated proteins in neurons.** *A* and *B*, N2a cells were transfected with either wild-type or mutant TLDc constructs as indicated and treated with 50 μm arsenite for 4 h. *A*, the cellular levels of *S*-nitrosylated proteins were quantified and compared with signal intensity in cells transfected with empty vector (*, *p* < 0.05; **, *p* < 0.01; ***, *p* < 0.001) or the corresponding wild-type construct (###, *p* < 0.001). Data are represented as the mean ± S.E. using one-way ANOVA with Dunnett's multiple comparison test or *t* test, respectively. *B*, representative images of cells stained for *S*-nitrosylated proteins after transfection with the Ncoa7-B constructs indicated and treated with arsenite. *Scale bar*: 100 μm.

We also quantified the level of protein *S*-nitrosylation under the same oxidative stress conditions. Protein *S*-nitrosylation levels were significantly decreased in cells overexpressing wild-type Ncoa7-B but not those transfected with Oxr1-C, Oxr1-FL, or Tbc1d24 (Fig. 6*A*). Importantly, mutations in Ncoa7-B that we have demonstrated are essential for the neuroprotective properties of the protein (for example, G93A and E216A; Fig. 4*B*) significantly increased the level of protein *S*-nitrosylation compared with cells transfected with wild-type Ncoa7-B (Fig. 6, *A* and *B*). Furthermore, cells overexpressing mutants of other TLDc proteins (Oxr1-FL E773A, Oxr1-C E293A, and Tbc1d24 A515V) showed significantly higher levels of protein *S*-nitrosylation than those transfected with the corresponding wild-type protein (Fig. 6*A*). These data suggest that TLDc proteins can modulate protein *S*-nitrosylation levels to protect neurons against oxidative stress.

##### Disruption of the TLDc Domain in Oxr1 Causes Neurodegeneration in Vivo

**FIGURE 7.**
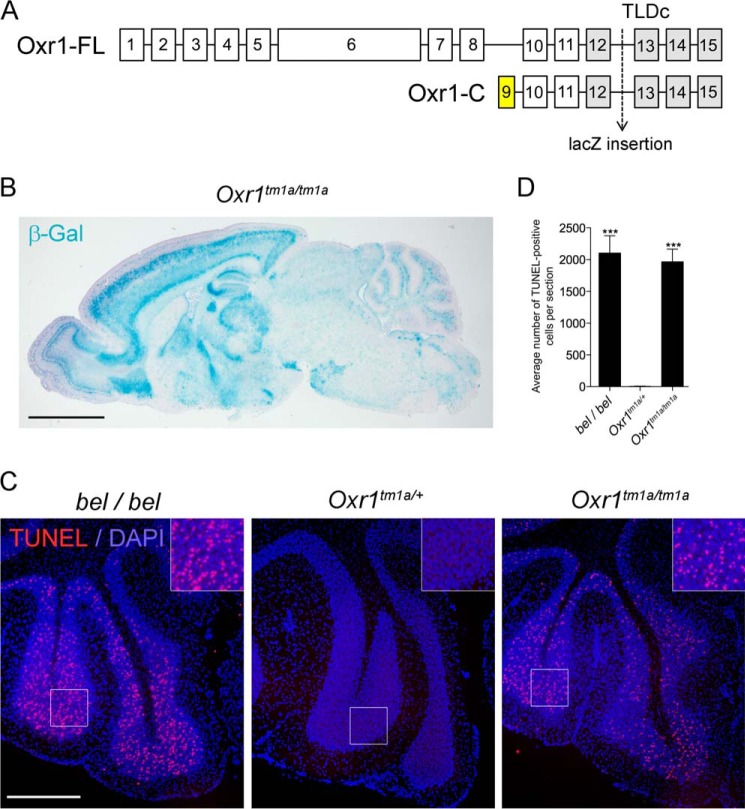
**The TLDc is necessary for neuronal survival *in vivo*.** *A*, schematic of mouse Oxr1-FL (based on accession number NM_130885) and Oxr1-C isoforms, showing the location of the LacZ insertion (for details see “Experimental Procedures”) that disrupts both isoforms of the protein by splicing-in from exon 12 (denoted as the tm1a allele of Oxr1). Note that exon 9 as shown is unique to Oxr1-C. Exons containing the TLDc domain are shown in *gray. B*, β-galactosidase staining of the brain of a mouse homozygous for the Oxr1 tm1a allele (*Oxr1^tm1a/tm1a^*). *C*, homozygous *Oxr1^tm1a/tm1a^* and *bella* (*bel/bel*) mice show a very similar pattern and distribution of TUNEL-positive (*red*) in the granule cell layer of the cerebellum at P21 compared with *Oxr1*^*tm1a*/+^ animals where no cell death was observed. These data are quantified in *D*. Data are represented as the mean ± S.E. using one-way ANOVA with Dunnett's multiple comparison test (***, *p* < 0.001). *Scale bars*: 1 mm in *B* and 400 μm in *C*. Higher (2×) magnification is shown in the *inset panels*.

We showed previously that a mouse deletion mutant (*bella*) lacking all isoforms of *Oxr1* and of the neighboring gene *Abra* displays ataxia from postnatal day (P) 20 and progressive apoptotic cell death in the cerebellar granule cell layer before death at approximately P26 (8). However, we wanted to specifically examine the disruption of the TLDc domain in the context of an *in vivo* mammalian system. Therefore, a new mouse mutant was generated in which the TLDc domain-coding exons shared by both Oxr1-FL and Oxr1-C were mutated by insertion of a LacZ reporter downstream of a splice acceptor site (Fig. 7*A*) (39). In this model any Oxr1 isoforms containing the TLDc domain will be truncated, losing the final 101 C-terminal amino acids. Homozygous mice carrying two copies of the mutant *Oxr1* allele (*Oxr1^tm1a/tm1a^*) were generated from heterozygous animals, and β-galactosidase staining of the brain at P18 (Fig. 7*B*) demonstrated an expression pattern almost identical to the *Oxr1 in situ* hybridization profile (Fig. 1*C*), as expected. In addition, we confirmed that expression of *Oxr1-FL* and *Oxr1-C* transcripts upstream of the insertion were present in the brain of *Oxr1^tm1a/tm1a^* animals (data not shown). Importantly, *Oxr1^tm1a/tm1a^* mutants displayed progressive cerebellar degeneration and ataxia to the same degree as the original homozygous *Oxr1* deletion mutants and over an identical timescale (Fig. 7, *C* and *D*). These data confirm that the TLDc domain is essential for Oxr1 function *in vivo*.

## Discussion

Here we present the first systematic functional investigation of the TLDc family of proteins performed in parallel. We demonstrate that each protein has the ability to protect neuronal cells from oxidative stress and that conserved residues and secondary structures within the TLDc domain are required for these important functional properties.

Since the original description of OXR1, it has been hypothesized that this protein plays an important role in oxidative stress resistance (7, 9). Evidence has since been accumulating that this protein is involved in a number of key stress response pathways that can be influenced by manipulating its expression (8, 10, 11, 13, 15, 43). Indeed, the importance of OXR1 and related TLDc domain-containing genes has been emphasized by the discovery that their disruption or deletion can result in a reduced lifespan in *Caenorhabditis elegans* (13), *Anopheles gambiae* (11), *Drosophila* (30), and mice (8) or cause a range of inherited human neurological disorders (20–22, 26, 44–46). Conversely, overexpression of TLDc proteins has been shown to increase life expectancy both in normal and disease-associated systems, for example in *Drosophila* (43) and mouse (14). Therefore, we focused this study on the TLDc domain as the significance of this highly conserved region has not been studied in a comparative manner to date.

We provide the first systematic expression data of TLDc domain-containing genes in embryonic and adult tissues, including *Kiaa1609* and *C20Orf118*, which have not been studied previously in mammalian systems. These data emphasized that each gene displays a distinct expression profile, although there are tissues and cell types where their expression is likely to overlap. It is also noteworthy that both the shortest TLDc domain-containing isoforms of *Oxr1* and *Ncoa7* are differentially expressed in certain tissues compared with the corresponding full-length genes; this is likely to be a reflection of their distinct promoters and potentially different functional requirements. For example, a shift in the splicing profile of human NCOA7, favoring *NCOA7-AS* (*Ncoa7-B* in mouse) over *NCOA7-FL*, is observed in macrophages stimulated with lipopolysaccharide, which models an immune insult (47). In addition, NCOA7-AS specifically is induced in interferon-β-treated peripheral blood mononuclear cells from healthy participants or patients with the autoimmune disease multiple sclerosis (41). There are several examples where independently regulated isoforms of a gene are functionally distinct, for instance only one particular variant of insulin-like growth factor 1 (*Igf1*) is beneficial in an *in vivo* model of ALS (48, 49) or is able to protect cardiomyocytes from oxidative stress insults (50). Indeed, we have shown previously that Oxr1-C has protein binding partners distinct from Oxr1-FL (15), further emphasizing the importance of studying independently the various TLDc domain-containing isoforms of this protein family.

As deletion of *Oxr1* causes decreased oxidative stress resistance in neuronal cells (8), we tested whether the same effect would be observed for *Ncoa7*, *Tbc1d24*, and *Kiaa1609* and found that a 54–64% knockdown of these three genes did not significantly increase sensitivity to oxidative stress. However, we demonstrated that an 82% knockdown of *Oxr1* is sufficient to render cells more susceptible to oxidative stress-induced degeneration. This is consistent with previous studies in cortical neurons lacking *Oxr1* (8). Moreover, we have shown previously that mice heterozygous for the *bella Oxr1* deletion, thus with only a partial reduction of *Oxr1* levels, were phenotypically normal (8). Taken together, this suggests there is a threshold level of TLDc gene expression below which other cellular mechanisms, or even other TLDc proteins, cannot compensate. Interestingly, the deregulation of the TLDc family has been demonstrated in several disease states, including an increase in OXR1 protein expression in end-stage ALS spinal cord biopsies (8) and a reduction in OXR1 in the posterior cingulate cortex of patients with Parkinson disease (51). In addition, levels of NCOA7 are reported to correlate with the clinical outcome of neuroblastomas (52). Thus, it appears that tight regulation of the TLDc domain-containing genes is a key feature of their neuroprotective properties; whether deregulation occurs at critical pre-symptomatic stages of disease warrants further investigation.

Our data suggest that the TLDc domain alone is able to confer oxidative resistance properties to all the TLDc members. This is in part corroborated by the observation that the shortest isoforms expressed in human and mouse, *Oxr1-C* and *Ncoa7-B*, are also functional in our assays despite containing almost exclusively the TLDc domain (8). A previous attempt to map the region of full-length OXR1 that confers oxidative stress resistance utilized a spontaneous mutagenesis assay in a DNA repair-deficient strain of *E. coli* (16). Human OXR1 deletion mutants were assessed, revealing that a particular exon of OXR1 (upstream of the TLDc domain, equivalent to exon 8; Fig. 7) encoded the anti-oxidant function. This apparent discrepancy with our current study could be explained by an organism-specific mode of action, although this is unlikely as human OXR1 has been shown to rescue a *S. cerevisiae OXR1* mutant phenotype, suggesting that there is functional conservation between species (9). The observations may, therefore, be due to the different methods used to assess oxidative stress resistance or that the two distinct regions or isoforms of OXR1 act via independent antioxidant mechanisms (16). For example, it has been proposed that the TLDc domain-containing proteins can influence the expression of key oxygen free-radical scavengers that, in turn, reduce the levels of ROS in the cell. Indeed, there is evidence that Oxr1 can modulate the expression of catalase (11), heme oxygenase (10), and glutathione peroxidase (8, 10, 11), potentially via a p21/NRF2-dependent pathway (10). Localization of OXR1 in the nucleus has also been described (53); however, which regions of the protein could directly or indirectly influence the transcription of these genes is still unknown.

It has also been hypothesized that the unique first coding exon of Ncoa7-B (NCOA7-AS in human) contains amino acid signatures of an aldo/keto reductase-like domain that could be responsible for the anti-oxidant properties of this short isoform (41). However, this hypothesis was based on a limited amino acid sequence alignment, whereas the entire aldo/keto reductase-like domain is predicted to be >300 amino acids long (54). Thus, we believe it is unlikely that the 25 amino acids of the Ncoa7-AS first exon could constitute or confer a functional enzymatic domain on its own.

Our own structural modeling combined with functional assays has highlighted for the first time the significance of certain highly conserved amino acids in the TLDc domain and how the context of this domain in the entire protein is critical. For example, mutating a conserved glycine residue (Gly-93 in Ncoa7-B; Figs. 3 and 4*B*) between α-helices 3 and 4 is highly detrimental to the oxidative stress resistance function of the domain (Fig. 3). Examining this position in more detail, the main chain torsion angles φ and ψ (Ramachandran angles) at this position are 102° and 179° (55); only a glycine residue is sufficiently flexible to accommodate these torsion angles, and therefore, the G93A mutation will likely compromise the correct placement of α-helices 3 and 4 during folding. We also focused on a C-terminal glutamic acid (Glu-216 in Ncoa7-B; Figs. 3 and Fig. 4*C*), as it is not only highly conserved in all TLDc proteins but it was also predicted to interact with a serine residue (Ser-60) bringing the N-terminal region of the TLDc domain into close proximity. We predict that the N-terminal region is the most flexible of the entire domain; therefore, it is likely that a mutation of this key glutamic acid residue would reduce the stability of the N-terminal region and displace one of the α-helices (*H1*, Fig. 4*C*). Indeed, mutations at this position were detrimental to the protective properties of the short isoforms of both Oxr1 and Ncoa7 but resulted in a surprising, almost complete loss-of-function in the full-length Oxr1-FL isoform. This suggests that allosteric interactions between the TLDc and other accompanying domains in this family of proteins may be significant.

Consistent with this hypothesis, compound heterozygous mutations in TBC1D24, one in the TBC domain and another in the TLDc domain, cause familial infantile myoclonic epilepsy (FIME), whereas the parents carrying either one of these mutations are phenotypically normal (21). Thus the TLDc mutation in this context must be deleterious as it is unable to compensate for the other mutant allele in the TBC domain. Previous cellular studies revealed that this TLDc mutation (A509V) did not influence the binding of TBC1D24 to ARF6; however, it was striking that the same epileptogenic TLDc amino acid change completely reverted the ARF6-dependent neurite outgrowth phenotype associated with TBC1D24 overexpression (21). Thus these data support the notion that the TLDc domain is essential for the normal function of TBC1D24. Moreover, we have shown here that the TLDc domain of Tbc1d24 can also elicit protection against oxidative stress and that mutations truncating Tbc1d24, as described in the most severe neurological conditions associated with this gene (22), abolishes this property.

In this study we focused on the protective properties of the TLDc family in neuronal cells; however, it has become apparent that TLDc proteins may also play an important role in infection and immunity (12, 14, 41, 56). For example, these proteins may protect against ROS that are generated as part of the normal immune response to infection (12). Furthermore, the therapeutic use of Oxr1 has also been tested successfully in a model of diabetic retinopathy (53) and in kidneys of a nephritis mouse model using Oxr1-overexpressing mesenchymal stem cells (57). Taken together, these data demonstrate that Oxr1 possesses an important and evolutionarily conserved protective function that could be exploited therapeutically in the future. In summary, our study provides important new functional insight into a family of proteins that contain the highly conserved TLDc domain and its vital role in oxidative stress protection and in a range of human neurological disorders.

## Author Contributions

M. J. F. performed and analyzed the experiments shown in Figs. 1, 2, 4, 5, and 6. P. L. O. performed and analyzed the experiments shown in Figs. 1 and 7. L. S.-P. analyzed and compiled the evolutionary and structural data in Figs. 3 and 4. K. X. L. performed the experiments shown in Fig. 7. M. J. F., K. E. D., and P. L. O. designed the experiments and wrote the paper. All authors reviewed the results and approved the final version of the manuscript.

## Footnotes

2 The abbreviations used are:

ROS: reactive oxygen species
*OXR1*: oxidation resistance 1
NCOA7: nuclear receptor coactivator 7
ARF6: ADP-ribosylation factor 6
qRT-PCR: quantitative real-time PCR
N2a: Neuro 2a
Bis-Tris: 2-[bis(2-hydroxyethyl)amino]-2-(hydroxymethyl)propane-1,3-diol
FL: full-length
TBC: Tre2/Bub2/Cdc16
AS: alternative start.
